# Comparative Effect of Different Nanoparticles with Different Concentrations on Fracture Toughness and Elastic Modulus of Restorative Dental Composite Resin

**DOI:** 10.3390/dj14030134

**Published:** 2026-02-28

**Authors:** Mohamed Ahmed Helal, Emad Amin Azmy, Amal Al-Faraj, Faris A. Alshahrani, Firas K. Alqarawi, Hamad S. AlRumaih, Mohammed M. Gad, Mostafa I. Fayad

**Affiliations:** 1 Department of Prosthodontics, Faculty of Dental Medicine, Al-Azhar University, Cairo 11651, Egypt; mhelal@azhar.edu.eg; 2 Prosthodontic Department, Faculty of Oral and Dental Medicine, Nahda University, New Beni Suef P.O. Box 62513, Egypt; 3 Department of Prosthodontics and Dental Implantology, College of Dentistry, King Faisal University, Al-Ahsa 31982, Saudi Arabia; 4 Department of Substitutive Dental Sciences, College of Dentistry, Imam Abdulrahman Bin Faisal University, P.O. Box 1982, Dammam 31441, Saudi Arabia; faalshahrani@iau.edu.sa (F.A.A.); fkalqarawi@iau.edu.sa (F.K.A.); hsalrumaih@iau.edu.sa (H.S.A.); mmjad@iau.edu.sa (M.M.G.); 5 Department of Substitutive Dental Sciences, College of Dentistry, Taibah University, Madinah 42314, Saudi Arabia; mifayad@taibahu.edu.sa

**Keywords:** composites, esthetic, nanoparticles, fracture toughness, modulus of elasticity

## Abstract

**Background/Objective:** Resin-based composite (RBC) gained wide popularity in dentistry due to its excellent biocompatibility, superior aesthetics, and good bonding to enamel and dentine. However, they have several shortcomings, including mechanical insufficiency and shrinkage tendency. Many researchers have utilized nanoparticles (NPs) as a reinforcing filler for RBCs. This article focused on assessing the impact of three different nanoparticles, ZrO_2_, TiO_2,_ and SiO_2,_ with concentrations of 3 wt% and 7 wt%, on the elastic modulus (E) and fracture toughness (K_IC_) of one commercial light-activated dental resin composite. **Methods:** 140 rectangular specimens were constructed according to ISO 4049 with dimensions (25 × 2 × 5 ± 0.03 mm) and (25 × 2 × 2 ± 0.03 mm) for fracture toughness and elastic modulus, respectively. Specimens were categorized into four main groups based on nanofiller types. Control: plain without filler (CC) and three modified ones with ZrO_2_ (ZC), TiO_2_ (TC), and SiO_2_ (SC). Furthermore, modified groups were divided into two subgroups according to nanofiller concentration, 3 and 7 wt% (ZC3, ZC7, TC3, TC7, SC3, and SC7), n = 10. Mechanical testing for fracture toughness was completed using a single-edge notched beam, while a three-point bending test was used for elastic modulus. Analysis of data was based on two-way ANOVA and Bonferroni post hoc (α = 0.05). **Results:** ZrO_2_ provided the most substantial improvement in both E and K_IC_, with the optimal performance observed at 3 wt% for stiffness and 7 wt% for toughness. TiO_2_ groups also enhanced these properties at both concentrations; however, the gains were less pronounced compared to ZrO_2_. SiO_2_ improved mechanical performance at 3 wt%, but a higher loading of 7 wt% resulted in reduced values. **Conclusions:** Resin-based composite modified with 3 wt% of NPs tends to possess higher fracture toughness and modulus of elasticity. Fracture toughness enhancement was concentration-dependent with ZrO_2_ NPs, where the best result was obtained with 7 wt%. Nanoparticle-reinforced composite, particularly ZrO_2_, may be suitable for prosthodontic applications.

## 1. Introduction

Although amalgam has long served as the standard choice for posterior restorations due to its durability and ease of placement, it is neither esthetically pleasing nor a suitable restoration under a removable partial denture (RPD). Moreover, there are potential adverse health effects due to the possible release of mercury. Accordingly, focus has shifted to resin-based composites (RBCs) [1]. RBCs have many significant advantages, including excellent esthetics, good chemical bond to tooth structures, and affordable cost. In addition to their adhesive properties and conservative tooth preparation, the widespread use of resin-based composites in anterior and other esthetically demanding areas is primarily driven by their ability to closely mimic natural tooth color, translucency, and surface gloss, allowing highly esthetic and minimally invasive restorations, but inadequate mechanical properties are still the main problem [2,3]. Regarding the improvement of inferior mechanical characteristics of RBCs, several attempts were made to incorporate different types of fillers such as glass fibers, ceramic particles, whiskers, and nanotubes, but some of them were not ideal due to poor filler–matrix bonding and large particle size, causing them to act as stress concentration areas [1,2].

Lately, the incorporation of nanoparticles (NPs) into RBCs has been used as a promising means of enhancing their properties because the tiny size of NPs (1–100 nm) plus a massive specific surface area contributes to unique characteristics compared to those observed in large-size particles, resulting in an ultimate success rate and long-term stability [4]. A lot of researchers’ articles have reported that the incorporation of nanotechnology could greatly increase the mechanical advantages of RBCs, such as fracture toughness (K_IC_), flexural properties, and wear resistance [5,6,7]. It was found that the type, content, and size of reinforcing fillers are important factors; Hua et al. assessed the effect of NPs and glass fibers and found that an improvement of elastic modulus by 30% requires 3% volume percentage of NPs, whereas achieving equivalent results required 6% volume fraction of glass fibers [8].

Zirconium oxide (ZrO_2_) NPs have garnered significant interest due to their exceptional characteristics of ultimate strength, abrasion resistance, and fracture toughness, enabling enhancements in hardness, stiffness, and resistance to crack propagation. Many researchers reported significant physical and mechanical improvement of RBCs by the addition of ZrO_2_ NPs [5,9,10]. Similarly, titanium oxide (TiO_2_) NPs have attracted attention, as they provide a wide range of positive properties, such as chemical stability, affordability, remarkable strength, and corrosion resistance, in addition to their antibacterial activity [5,11]. Elsaka et al. reported improved fracture toughness of glass ionomer filled with 3 and 5 wt% of TiO_2_ NPs [12]. Silicon oxide (SiO_2_) NPs occupy a superior position in scientific research because of their easy preparation, excellent mechanical properties, good electrical insulation, and compatibility with resin matrices. Azmy et al. and many other researchers reported that the incorporation of SiO_2_ NPs into RBCs would increase their mechanical properties [5,6,13]. In prosthetic dentistry, the addition of SiO_2_ NPs to the polymethylmethacrylate improved its abrasion resistance with high color stability [5,14].

The fracture toughness (K_IC_) is represented by the energy absorbed by a material before crack propagation. K_IC_ is a highly recommended method to assess the fracture resistance of biomaterials; restoration with high fracture toughness will better tolerate high occlusal stresses with improved clinical service [15]. Hosseinalipour et al. stated that K_IC_ of Bis-GMA/TEGDMA resin composite significantly increased when the inorganic filler particles increased to 40 wt%, denoting the worth of inorganic filler percentage in improving the mechanical advantage of dental composite [16]. The elastic modulus is defined as the slope of the linear (elastic) portion of the stress–strain curve; it is usually correlated with hardness, making it a useful additional indicator of wear resistance. It is directly related to the extent of deformation under external loading; thus, posterior dental composites require a sufficient modulus to resist high masticatory loads. Furthermore, the elastic modulus of dental restorative materials should approximate that of enamel and dentin to ensure improved stress distribution [2,17]. A study completed by Prabhakar et al. showed moderate enhancement in elastic modulus and fracture toughness by 10–15% with the addition of 4 wt% nanohydroxyapatite [18].

The application of RBCs in dentistry varies from simple restoration to the construction of onlays, inlays, veneers, crowns, provisional restorations, and denture teeth. Denture teeth should exhibit superior wear resistance for maintaining adequate chewing efficiency and to avoid decreasing the properly determined vertical dimension of occlusion. Insufficient wear resistance was still a major demerit of acrylic denture teeth, so newly developed nanocomposite teeth demonstrated significantly higher wear resistance than other types of acrylic denture teeth [19,20].

RPDs are mainly retained by mechanical means in the form of placing clasps into suitable undercuts on the abutment teeth, but in many situations, a natural undercut cannot be located by surveying and must be created artificially via crowns or by recontouring with RBC; it was reported that recontouring of abutment tooth using RBCs is a reliable, conservative, and simple method for creating sufficient undercuts for clasp engagement [21]. Helal et al. found an insignificant effect in the degree of retentive force between natural enamel, dental resin composite, and CAD/CAM ceramics [22].

Although many studies have examined individual NP types, limited studies have directly compared the effect of different nanofillers at multiple concentrations on the properties of RBCs. In this context, the current in vitro research investigated the influence of ZrO_2_, TiO_2_, and SiO_2_ NPs at 3 wt% and 7 wt% on the E and K_IC_ of one commercial light-cured restorative composite. The null hypothesis stated that neither NP type nor concentration would significantly affect these mechanical properties.

## 2. Materials and Methods

Types, chemical ingredients, and manufacturers’ specifications of involved materials in the research are summarized in Table 1. All materials were purchased from scientific chemical and dental suppliers, where specimens were prepared in the Prosthodontics Department, Nahda University, and data were obtained from laboratory-based studies. Sample size calculation was completed using a power analysis test based on prior studies; the power was set at 0.80 (1 − β) with a significant level of 0.05, so 140 specimens were prepared (n = 10, 70/test) [5,20,23].

**Figure 1:**
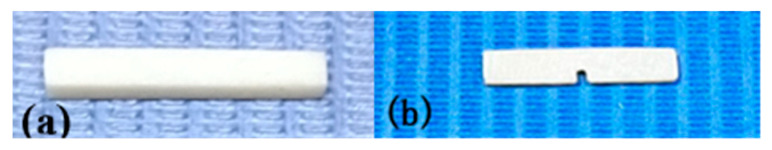
Composite resin specimens for E (**a**) and K_IC_ (**b**) tests.

Based on ISO 4049:2000 guidelines and previous studies [24], rectangular specimens were constructed by one examiner to diminish any interpersonal variability in specific dimensions per test (25 × 2 × 2 ± 0.03 mm and 25 × 2 × 5 ± 0.03 mm for modulus of elasticity and fracture toughness, respectively); see Figure 1 [15,16,17,18,19,20,21,22,23,24,25]. Specimens were apportioned into four principal groups based on the nanofiller types: one control (without filler incorporation) and 3 reinforced groups (ZrO_2_, TiO_2_, and SiO_2_). Moreover, each reinforced group was further subdivided based on nanofiller concentrations into 2 subgroups (3 wt% and 7 wt%); see Table 2.

Surface modification of NPs was completed for each one by utilizing a silane coupling agent [3-TMSPM] to promote functional groups required to chemically link the inorganic NPs with the organic matrix, allowing better bonding between them. Acetone was used for dissolving TMSPM to evenly coat NPs surfaces that were incorporated into the acetone/TMSPM solution and agitated by a magnetic stirrer (HS-350C, Hangzhou Yooning Instrument Co., Ltd., Hangzhou, China) for 1 h. Solvent elimination was completed under vacuum utilizing a machined evaporator (Rotavapor^®^ R-300, Buchi AG, Flawil, Switzerland) for 30 min with 150 rpm at 60 °C. Later, the dried mixture was heated at 120 °C for 2 h followed by bench-cooling until ambient temperature [14]. At this moment, surface-treated nanoparticles were obtained.

A sufficient quantity of silane coupling agent (X), which is needed to obtain a uniform and efficient coating of NPs, was obtained from the following formula [26]:

X = (A/ω) ƒ

where A: NP surface area (m^2^/g), ƒ: NPs mass (g), and ω: surface coverage of silane per gram (2525 m^2^/g)

The crystalline phases of treated NPs were confirmed via X-ray diffraction (XRD) through Cu-Kα radiation (30 mA, 40 kV, 1200 W), with scan range 10–80° 2θ, step size 0.05°, and step time 1.5 s [27].

**Figure 2:**
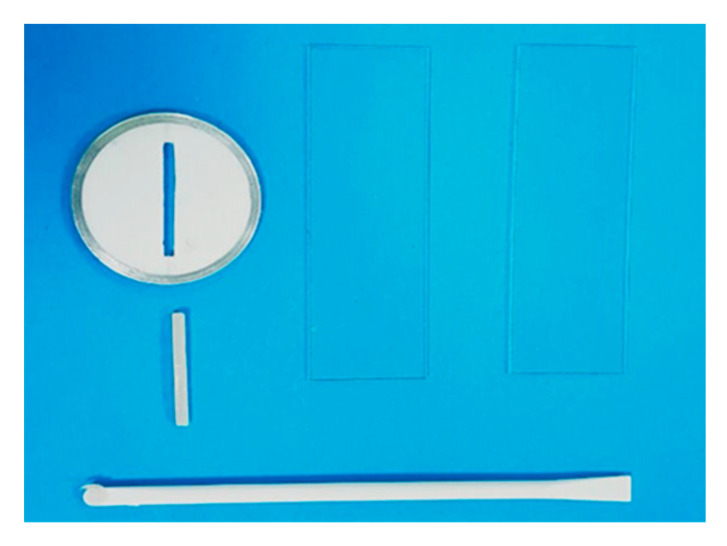
Split Teflon molds for specimen’s fabrication using composite resin.

A custom-made Teflon split mold (Figure 2) was constructed with the previously mentioned dimensions for each test to be used for specimens’ fabrication. Teflon split mold consists of two parts with an outer metallic ring to assist reassembling of the two parts together; mold was placed over a glass slide covered with a celluloid Mylar strip (Henry Schein Inc., Melville, NY, USA). An adequate number of surface-treated NPs was weighed utilizing a digital balance (Denver Instrument, Goettingen, Germany) of 0.001 g accuracy and then mixed separately with two concentrations (3 and 7 wt% of RBC), as described in Table 3.

Prior to mixing of NPs with RBCs, the composite syringes were preheated using an AR composite heater (Active Resin Heater; Foshan M&Y Medical instrument Co., Ltd., Foshan, Guangdong, China); the heater was turned on for 15 min, and then the syringes were completely inserted in the heater chamber and kept for 10 min to reach a temperature of 60 ± 4 °C [28].

On a glass slab, preheated RBC material was hand-mixed with modified NP powder separately by a stainless-steel spatula under daylight for 5 min until a uniform color and homogeneous mixture was gained [5,29]. Only one investigator made this step with a standardized manipulation method, including the time and direction of spatulation and surface area for spreading during spatulation to decrease variability as much as possible.

RBC was condensed increment by increment into the mold with slight overfilling, covered by a Mylar strip (KerrHawe SA No686, Bioggio, Switzerland) and a glass slide, and then compressed for 30 s to extrude any excess material, which was eliminated using a cement spatula. The specimens were photo-polymerized at the central and peripheral areas of each specimen from both top and bottom using an LED unit (DTE O-Light Max dental wireless LED curing light, Guilin Woodpecker Medical Instrument Co., Ltd., Guilin, China), which emits 1000 mW/cm^2^ irradiance, verified with a built-in radiometer for 20 s per surface. Based on manufacturers’ instructions, the curing tip was placed at zero distance and perpendicular to the specimens’ surfaces to ensure perfect photoactivation of specimens. After that, Mylar strips were discarded, the molds were dismantled, the specimens were extruded, and an additional curing shot was applied to all sides [17].

Specimens were finished utilizing Sof-Lex™ aluminum-oxide discs (Minnesota Mining and Manufacturing Company Oral Care, St. Paul, MN, USA), and polishing was completed carefully with sand paper with a sequence of 400, 600, and 800 grit (Minnesota Mining and Manufacturing Company Oral Care, St. Paul, MN, USA) mounted on rotary unit (MetaServ 250 Grinder-polisher, Buehler Ltd., Lake Bluff, IL, USA) running at 250 rpm under continuous water cooling for 30 s for each procedure, and the disc was replaced after triple applications to gain a glossy surface [5]. Finally, cleaning was performed for 2 min in an ultrasonic bath (Transonic T 460, Camlab Ltd., Cambridge, UK) to remove any rubbish attached to specimens.

Optical inspection of finished specimens was performed to detect any surface defects; specimens with voids, porosities, warpage, or broken edges were discarded. Dimensions were confirmed with an electronic digital caliper (TOC Dental; Orthodontic Company Ltd., Fareham, Hampshire; 0.01 mm accuracy) at the specimens’ center and at two different points. Prior to testing, specimens were placed in plastic containers containing distilled water for 48 h at 37 °C based on ADA guidelines to simulate oral conditions [25].

The elastic modulus was measured via a three-point bending test (3 Pb) on a universal testing machine (LRX Plus, Ametek, Fareham, UK; load cell 5 kN) with a span length (L = 20 mm) and crosshead speed of 0.75 mm/min. An individual specimen was mounted horizontally in a loading fixture with the aid of a jig. Load was adjusted at zero then increased progressively until fracture occurred; specimen deflection (mm) and peak load (N) were recorded, and then elastic modulus (E) was calculated from the following formula [24]:

E = (PL^3^)/(4bh^3^ d)

where E = elastic modulus (MPa), P = peak load causing fracture (N), L = length between two supports (mm), b and h = specimen width and thickness respectively (mm), and d = specimen deflection at the fracture point (mm).

A single-edge notched test was used using a 3 Pb testing unit for fracture toughness investigation. Standardized rectangular specimens, 25 × 2 × 5 ± 0.03 mm, were constructed as previously described, and then a piercing notch at half of the specimen’s width, 2.5 mm, was fabricated by machining a prepared slot in the middle of the Teflon mold. Then, the radius of the main notch at its bottom was measured with a sharp blade and a gentle tapping by one investigator. The crack length and sharpness were confirmed for all specimens utilizing a stereomicroscope (EMZ-5; Meiji Techno Co., Ltd., Saitama, Japan) at 1.5× magnification for standardization. At the center of the notched specimen, a load was applied using a 3 Pb machine, with a 20 mm length between both supports and a crosshead speed of 0.5 mm/min. When the fracture point was reached, the peak force causing fracture was listed, and K_*IC*_ was calculated using the following equation [24,30]:

K_IC_ = [*PL*/*BW*^1.5^] × *Y*

where K_IC_ = the fracture toughness (MPa√m), P = load causing fracture (N), L = length between both supports (mm), B and W = specimen thickness and width (mm), (a) = notch length (mm), and Y = geometry value, obtained from the following equation [30]:

Y = [2.9 (a/w) ^1/2^ − 4.6 (a/w) ^3/2^ + 21.8 (a/w)^5/2^ − 37.6 (a/w)^7/2^ + 38.7 (a/w)^9/2^]

Data were collected, tabulated, and entered in software program (IBM SPSS for Windows, Version 23.0, IBM Corp. Armonk, NY, USA) for statistical analysis. Normality was verified utilizing the Shapiro–Wilk test. Homogeneity of variance was assessed utilizing Levene’s test; no significant violations were detected (*p* > 0.05) for all comparisons, so conventional two-way ANOVA was applied for data analysis (factors: NP type, concentration, and their interaction) followed by Bonferroni post hoc tests (*α* ≤ 0.05). Effect sizes (η^2^) were reported.

## 3. Results

Numerical data presented a normal (parametric) distribution. Two-way ANOVA revealed statistically significant main effects for NP type (*p* < 0.001) and NPs concentration (*p* < 0.001) on the E, while the interaction between the two variables was statistically non-significant (*p* = 0.761), indicating independent contributions of nanoparticle type and concentration (Table 4).

Mean E values for all experimental groups, except TC7 and SC7, were significantly higher than the plain composite (CC) (*p* < 0.001) (Table 5). The highest E value was recorded with ZC3 (7.2 ± 0.84 GPa), followed by SC3 (6.38 ± 0.53), TC3 (5.79 ± 0.34), and ZC7 (5.61 ± 0.72) groups, all above the control (4.58 ± 0.53). TC7 (4.42 ± 0.44) and SC7 (4.77 ± 0.17) did not differ from control.

Overall, the ZC group outperformed other groups with no significant difference between the TC and SC groups. Within each NP type, the 3 wt% concentration yielded higher modulus values compared to 7 wt% (Table 6).

Two-way ANOVA analysis of fracture toughness (K_IC_) results showed that NP type, concentrations, and their interaction showed a significant impact on K_IC_ (*p* < 0.001), suggesting a combined influence of both factors (Table 7).

All experimental groups demonstrated significant enhancement in K_IC_ except TC7 and SC7. The highest K_IC_ was obtained in group ZC7 (2.65 ± 0.16, ~55% above control), which was significantly higher than all other groups (*p* < 0.001). ZC3 ranked second (2.41 ± 0.19), followed by SC3 (2.29 ± 0.19), both significantly higher than TC3 (2.15 ± 0.15). TC7 (1.82 ± 0.16) and SC7 (1.81 ± 0.11) showed no significant improvement (*p* > 0.05) (Table 8). At 3%, ZrO_2_ and SiO_2_ produced similar toughness (*p* = 0.17), both outperforming TiO_2_. At 7%, only ZrO_2_ showed a positive concentration effect, while TiO_2_ and SiO_2_ decreased slightly (Table 9).

## 4. Discussion

Since the mid-1960s, RBCs have become one of the most preferred restorative biomaterials in esthetic dentistry and many applications in prosthetic dentistry, but mechanical limitations such as elastic modulus and fracture toughness (crack propagation resistance) can result in premature failure during clinical practice [4,15].

To overcome these shortages, a lot of studies have been conducted to develop recent materials or improve existing ones to meet the ideal requirements for restorative material; incorporation of NPs into composite resins has emerged as a promising approach to improve their physical and mechanical properties for better durability and performance, so they have become an interesting topic for research [16,18]. Based on the present results, the incorporation of SiO_2_, ZrO_2_, and TiO_2_ significantly improved the modulus of elasticity and fracture toughness of RBCs, so the supposed null hypothesis was rejected.

The novelty of the present work comes from the deficiency of published articles regarding the comparison between the effect of different NPs with different concentrations on the elastic modulus and fracture toughness of light-cured RBCs. Based on the most widespread NPs assessed in previous research, in addition to their better biomechanical properties, three different NPs, ZrO_2_, TiO_2_, and SiO_2_, were selected because of their documented enhancement of the mechanical properties of polymeric materials, besides their antibacterial effect [5,11]. The optimal concentrations of NPs that can cause a significant improvement of RBC properties are crucial; based on the most popular concentrations assessed in the former articles, two concentrations, 3 wt% and 7 wt%, of NPs were selected because high concentrations may cause NPs agglomeration, leading to weakening the resultant composite. Furthermore, this may give rise to a massive color change in the reinforced nanocomposite [5,7].

As the higher inorganic filler content of commercial composite resin leads to a higher viscosity, preheating composite resin from 50 to 70 °C has been utilized to enhance the flowability of regular consistency composites and make them smoother, easier to manipulate, and improve their adhesion to the added filler; Poubel et al. and others supported that heating of composite resin for 10 min at 60 ± 4 °C results in reducing its viscosity by at least 84% [28,31].

According to ISO 4049, Type 1, modulus of elasticity and flexural strength were utilized as a specific test method for approving the polymer-based materials. The three-point bending test is the most common method for measuring elastic moduli, while a single edge notch (SENB) is widely accepted for fracture toughness evaluation, precise specimen construction, notch production, and controlled testing parameters, as load and specimen geometry are critical for reliable data [23,24].

Split mold was used during specimens’ fabrication, so that no force was applied during specimen removal to avoid any deformation. Also, each specimen was polymerized from the top and both sides after disassembling the mold to achieve optimal curing depth and quality [5].

The present article showed a variable effect among investigated NP types and concentrations on the modulus of elasticity of RBCs; the maximum E value was observed with 3 wt%, with a high record owing to ZrO_2_ NPs. ZrO_2_ with 7 wt% causes a significant increase in E values; this result may be related to the homogenous distribution of ZrO_2_ NPs, which permits them to fill micro-gaps within the polymer chains, leading to restriction of their mobility with improved elastic modulus. In addition, the key to improving nanocomposites was the surface treatment by salinization of NPs, which led to enhancing matrix–filler interfacial adhesion and good dispersion within the resin matrix [32,33].

Significant enhancement of E with both concentrations of ZrO_2_ is in line with Sadeghi et al., who found an enhancement of the modulus by 20% via adding 3–5% of ZrO_2_ NPs and linked this improved stiffness to homogeneous dispersion and high E of ZrO_2_ itself (~200 GPa) [34]. Also, a study completed by Abdel Hafez et al. confirmed the enhancement of mechanical properties of RBCs with a high concentration of ZrO_2_, indicating that this improvement was concentration-dependent [35]. In disagreement with our findings, Rafid et al. reported that 1 and 3 wt% of ZrO_2_ provide a positive effect on the physicochemical properties of resin composite, whereas high concentration (5, 7, or 10 wt%) had an adverse effect [23]. This disagreement may be attributed to differences in methodology and usage of different brands of NPs.

The present outcomes showed a significant increase in E with low concentration (3 wt%) of TiO_2_ and SiO_2_ NPs, in accordance with Dafar et al., who found a significant improvement in E with the incorporation of 3% TiO_2_, 58% greater than the unmodified resin composite [29]. Also, Souza et al. concluded that adding 1.5% TiO_2_ significantly increases the elastic modulus of the experimental composite [36].

Chen et al. incorporated SiO_2_ (20–50 nm) into methacrylate-based dental resins and reported a significant improvement in elastic modulus and fracture toughness, up to 30% at 5 wt% filler loading [37]. On the other hand, the findings of this research were at variance with Hosseinalipour et al., who found mechanical enhancement of the nanocomposite with high concentrations of SiO_2_ (20–50 wt%) [16]. Additionally, Garoushi et al. recorded a passive effect on the modulus and flexural properties of SiO_2_ nanocomposite [38]. The disagreement noted above may be referred to different commercial materials and methodologies and different NPs sizes.

In the current article, an enhancement in E with both tested concentrations of ZrO_2_ was found, whereas insignificant improvement resulted with high concentrations of SiO_2_ and TiO_2_, which may be referred to different particle sizes and inherent properties of each NPs used; in addition, this drop in E values with 7% TiO_2_ and SiO_2_ could be attributed to NPs agglomeration with poor dispersion between filler and organic matrix [5,38].

Fracture toughness (K_IC_) of RBC biomaterials is considered a good indicator for their clinical performance. Based on the present findings, a significant increase in K_IC_ with different types and concentrations of NPs, which is in accordance with many authors who reported that NP addition to RBCs is a validated strategy to significantly improve their mechanical properties, including modulus of elasticity and fracture toughness with overall durability [16,17,29,30].

The present study typically reported values of 1.7, 2.53, 1.99, and 2.05 for control, ZiO_2_, TiO_2_, and SiO_2_ groups, respectively, with no difference between TiO_2_ and SiO_2_; both presented the same effect. The obtained K_IC_ values of all investigated groups fall within the high ranges reported by a previous study by Ilie et al. (0.8–2 MPa·m^0.5^) [15].

The present work found that the increase in K_IC_ is directly proportional to the increase in the NP concentration, where the maximum K_IC_ was recorded with 7% ZrO_2_. This may be attributed to their high hardness and the transformation toughening of ZrO_2_, which can deplete the energy of microcracks and arrest crack propagation. This finding aligns with several authors who reported that heavier filler content would lead to an improvement in K_IC_ of RBCs [12,30]. The outcomes of the current research reported an enhancement in K_IC_ with the incorporation of 3% of TiO_2_ and SiO_2_. In line with this result, Li et al. found that 3 wt% of TiO_2_ NPs enhanced K_IC_ up to 25% and attributed this to crack deflection and energy dissipation at nanoparticle interfaces [39]. Also, Dafar et al. concluded that 3% functionalized n-TiO_2_ significantly increased the K_IC_ value by 36% [29]. Furthermore, Elsaka et al. found that 3–5 wt% TiO_2_ had improved K_IC_ of glass ionomer [12]. Tian et al. recorded a valuable improvement in the flexural properties of RBCs through the addition of SiO_2_ particles [13]. Against the present results, a study conducted by Hosseinalipour et al. noticed a remarkable improvement in the K_IC_ of Bis-GMA/TEGDMA composite modified with a high concentration of SiO_2_ [16]. This difference in results may be attributed to different inherent properties of the investigated commercial composite brands and different methodologies, such as curing protocol or testing conditions. As K_IC_ evaluation is highly sensitive to the specimens’ geometry, notch length, and load cell, it makes it difficult to perform an accurate comparison between different research. It was found that the NP type, size, and their own properties are critical factors in the achieved improvement, which explained why NPs behave differently [8].

From the clinical point of view and clinical application, related to composite limitations as a rest seat under RPD in comparison to other materials, which has already been reported in the literature under the category of surveyed ceramic crown, previous studies reported that composite could be used as a rest seat for RPD; however, they have limitations regarding fracture incidence of the composite [40,41]. Based on the findings of this study, the reinforced composite could be clinically suitable and overcome the pure composite limitation. On the other hand, caution was mandatory during the selection of a suitable type and percentage of NPs, which makes us appreciate the optical and mechanical properties of RBCs; therefore, this point will remain a primary objective for researchers, so further investigations are required to compare the reinforced composite with CAD-CAM ceramic materials that are commonly used in prosthodontics to prove the applicability of reinforced composite.

The main limitations of this research include the absence of thermocycling, SEM analysis, the usage of only one commercial brand of composite with one shade, an in vitro design, and a focus on static mechanical properties only. Fatigue resistance, wear behavior, and long-term water sorption effects were not assessed. Despite applying the Bonferroni correction to control for Type-I error inflation, conducting multiple comparisons across the study still carries a residual risk of Type-I error inflation. Future studies should be directed toward evaluating the impact of nanoparticle incorporation on optical properties, degree of conversion, polymerization shrinkage, and biocompatibility to ensure comprehensive clinical applicability in correlation between both mechanical and optical outcomes. In addition, further research using aging protocol, SEM, and TEM analysis to assess the dispersion quality of the reinforced composite with multiple brands of composites and different NP concentrations will be needed.

## 5. Conclusions

Concerning the present study’s findings and under its limitations, the following conclusions were made:

1. Composite resins modified with metal oxide NPs significantly enhance the E and KIC. ZrO_2_-modified composites show strong potential for high-stress clinical applications, while TiO_2_ and SiO_2_ have the same degree of enhancement.
2. ZrO_2_ produced the greatest improvements, with 3 wt% yielding the highest stiffness and 7 wt% yielding the highest toughness.
3. TiO_2_ and SiO_2_ at 3 wt% also improved mechanical properties, but increasing to 7 wt% offered no added benefit.
4. Within the range of tested concentrations (3 and 7%), the toughening effect was concentration-dependent for ZrO_2_ but plateaued for TiO_2_ and SiO_2_.

## Figures and Tables

**Table 1:** Composition and key properties of the materials used in the study.

Trade Name	Manufacturer	Specifications
Any-Com^®^	MEDICLUS Co., Ltd., Gwangju, Republic of Korea	Radiopaque, packable, nanohybrid Zirconium composite resin, with medium to high viscosity (about 10^4^–10^6^ Pa·s). Resin: methacrylate base-modified resin (Bis-GMA/TEGDMA) 30%. Fillers: 0.01–2.5 µm Zirconium filler 70% light cured, A2 shade. Lot AC45T623, Exp 12-May-2027
ZrO_2_ nanoparticles	NanoGATE, Ciro, Egypt	White spherical, tetragonal nanoparticles with average size 12 ± 3 nm, purity > 99%, density 5.89 g/cm^3^ at 25 °C
TiO_2_ nanoparticles	NanoGATE, Ciro, Egypt	White spherical and anatase particles with average size 15 ± 3 nm, purity > 99%, density 4.26 g/cm^3^ at 25 °C.
SiO_2_ nanoparticles	NanoGATE, Ciro, Egypt	White spherical, and amorphous particles with average size 21 ± 3 nm, purity > 99%, density 2 g/cm^3^ at 25 °C.
Silane coupling agent	Sigma-Aldrich Chemie GmbH Riedstrasse2, Steinheim am Albuch, Germany	Ethanol 99.7%, 3-trimethoxysilyl propyl methacrylate, 97% (γ-MPS) silane, purity 98%. Lot no. 440,159

**Table 2:** Grouping and coding of experimental variables.

	Group	Code	Description
Group 1	Control	CC	Unmodified light-cured RBCs.
Group 2	ZrO_2_	ZC3	Light-cured RBCs modified with 3 wt% of ZrO_2_ NPs
		ZC7	Light-cured RBCs modified with 7 wt% of ZrO_2_ NPs
Group 3	TiO_2_	TC3	Light-cured RBCs modified with 3 wt% of TiO_2_ NPs
		TC7	Light-cured RBCs modified with 7 wt% of TiO_2_ NPs
Group 4	SiO_2_	SC3	Light-cured RBCs modified with 3 wt% of SiO_2_ NPs
		SC7	Light-cured RBCs modified with 7 wt% of SiO_2_ NPs

**Table 3:** Mixing proportions for nanoparticle-modified composites.

Concentration of NPs	Weight of Composite	Weight of NPs
0 wt% (control)	15 g	0 g
3 wt%	15 g	0.45 g
7 wt%	15 g	1.05 g

**Table 4:** Two-way ANOVA results for effects of NP types and concentration on elastic modulus (GPa).

Source of Variation	Type III Sum of Squares	df	Mean Square	*F*-Value	*p*-Value	Effect Size (Partial Eta Squared)	95% CI
Filler type	14.855	2	7.428	24.539	<0.001 *	0.438	0.241–0.564
Concentration	32.362	1	32.362	106.917	<0.001 *	0.629	0.474–0.722
Filler type × Concentration interaction	0.166	2	0.083	0.275	0.761	0.009	0–0.073

* Significant at *p* ≤ 0.05, (df) degrees of freedom = (n − 1).

**Table 5:** Mean values, standard deviation (SD) of elastic modulus (GPa), and pairwise comparisons for different groups and outcomes for effects of both variables (type and concentration).

Variables		Mean ± SD	*p*-Value	Effect Size (Partial Eta Squared)	95% CI
Filler type	CC	4.58 ± 0.53 ^C^	<0.001 *	0.438	0.241–0.564
	ZC	6.31 ± 1.05 ^A^			
	TC	5.11 ± 0.8 ^B^			
	SC	5.57 ± 0.91 ^B^			
Concentration	0%	4.58 ± 0.53 ^B^	<0.001 *	0.629	0.474–0.722
	3%	6.40 ± 0.77 ^A^			
	7%	4.93 ± 0.70 ^B^			

* Significant at *p* ≤ 0.05. Different capital letters within each variable indicate statistically significant differences.

**Table 6:** Mean ± SD of elastic modulus for each nanoparticle type at each concentration (interaction means).

Concentration	CC	ZC	TC	SC	*p*-Value	Effect Size (Partial Eta Squared)	95% CI
	Mean ± SD	Mean ± SD	Mean ± SD	Mean ± SD			
CC	4.58 ± 0.53	4.58 ± 0.53 ^a^	4.58 ± 0.53 ^a^	4.58 ± 0.53 ^a^	-	-	-
3%	4.58 ± 0.53 ^D^	7.02 ± 0.84 ^A,b^	5.79 ± 0.34 ^C,b^	6.38 ± 0.53 ^B,b^	<0.001 *	0.283	0.098–0.428
7%	4.58 ± 0.53 ^C^	5.61 ± 0.72 ^A,c^	4.42 ± 0.44 ^B,a^	4.77 ± 0.17 ^B,a^	<0.001 *	0.282	0.098–0.427
*p*-value	-	<0.001 *	<0.001 *	<0.001 *			
Effect Size (Partial Eta Squared)	-	0.344	0.332	0.407			
95% CI	-	0.160–0.491	0.150–0.481	0.220–0.545			

* Significant at *p* ≤ 0.05. Different capital letters per row (horizontally) indicates statistically significant difference between NP types. Different small letters per column (vertically) indicate statistically significant difference between concentrations.

**Table 7:** Two-way ANOVA findings for effects of nanoparticle type and concentration on K_IC_.

Source of Variation	Type III Sum of Squares	df	Mean Square	*F*-Value	*p*-Value	Effect Size (Partial Eta Squared)	95% CI
NP type	3.497	2	1.748	73.571	<0.001 *	0.7	0.560–0.772
Concentration	0.525	1	0.525	22.079	<0.001 *	0.26	0.092–0.416
NP type × Concentration interaction	1.43	2	0.715	30.091	<0.001 *	0.489	0.296–0.605

* Significant at *p* ≤ 0.05, (df) degrees of freedom = (n − 1).

**Table 8:** Mean values, standard deviation (SD), and pairwise comparisons of fracture toughness for different groups and findings for main effects of both variables.

Variables		Mean ± SD	*p*-Value	Effect Size (Partial Eta Squared)	95% CI
NP type	CC	1.70 ± 0.09 ^C^	<0.001 *	0.7	0.560–0.772
	ZC	2.53 ± 0.21 ^A^			
	TC	1.99 ± 0.22 ^B^			
	SC	2.05 ± 0.29 ^B^			
Concentration	0%	1.7 ± 0.09 ^C^	<0.001 *	0.26	0.092–0.416
	3%	2.28 ± 0.20 ^A^			
	7%	2.09 ± 0.42 ^B^			

* Significant at *p* ≤ 0.05. Different capital letters within each variable indicate statistically significant differences.

**Table 9:** Mean and standard deviation (SD) of fracture toughness for each nanoparticle type at each concentration (interaction means).

Concentration	CC	ZC	TC	SC	*p*-Value	Effect Size (Partial Eta Squared)	95% CI
	Mean ± SD	Mean ± SD	Mean ± SD	Mean ± SD			
Control	1.7 ± 0.09	1.7 ± 0.09 ^a^	1.7 ± 0.09 ^a^	1.7 ± 0.09 ^a^	-	-	-
3 wt%	1.7 ± 0.09 ^C^	2.41 ± 0.19 ^A,b^	2.15 ± 0.1 ^B,b^	2.29 ± 0.1 ^A,b^	0.002 *	0.182	0.031–0.329
7 wt%	1.7 ± 0.09 ^C^	2.65 ± 0.1 ^A,c^	1.82 ± 0.1 ^B,a^	1.81 ± 0.1 ^B,a^	<0.001 *	0.754	0.635–0.813
*p*-value	-	0.001 *	<0.001 *	<0.001 *			
Effect size (Partial Eta squared)	-	0.162	0.26	0.432			
95% CI	-	0.030–0.320	0.092–0.416	0.246–0.566			

* Significant at *p* ≤ 0.05. Different capital letters per row (horizontally) indicate statistically significant difference between NP types. Different small letters per column (vertically) indicates statistically significant difference between concentrations.

## Data Availability

The data presented in this study are available upon reasonable request from the corresponding author.
